# Supplementary material for: Specific binding of GPR174 by endogenous lysophosphatidylserine leads to high constitutive Gs signaling
Source: Nat Commun. 2023 Sep 22;14:5901. doi: 10.1038/s41467-023-41654-3 (PMC10516915; doi:10.1038/s41467-023-41654-3)
Supplement: Supplementary file 1 — Supplementary Information [file 41467_2023_41654_MOESM1_ESM.pdf]

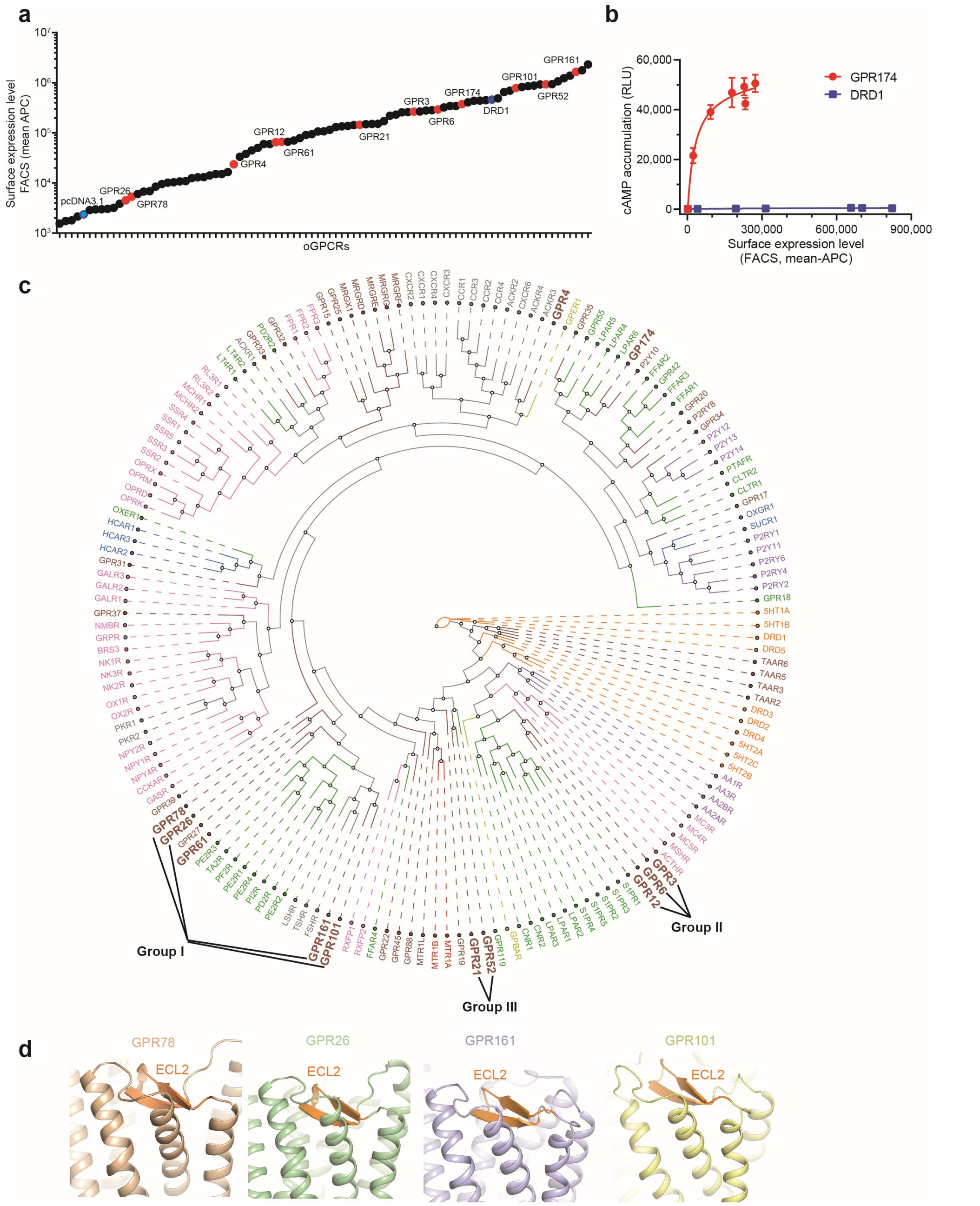

Supplementary Fig. 1 | The caoGPCRs are closely related to GPCRs known to be activated by lipids.

a, The surface expression levels of oGPCRs were determined by FACS using fluorescence-conjugated anti-Flag antibody.

b, cAMP accumulation level in Expi293F cells transfected with increasing concentration of GPR174 or D1R. Bar graphs represent mean ± SEM from three independent experiments. Source data are provided as a Source Data file.

c, Phylogenetic tree of GPCRs including caoGPCRs and representative members of class A GPCR subfamilies.

d, Structures of GPR78, GPR26, GPR161 and GPR101 predicted by Alphafold reveal a conserved β-haripin structure of ECL2 that is buried in the orthosteric binding pocket.

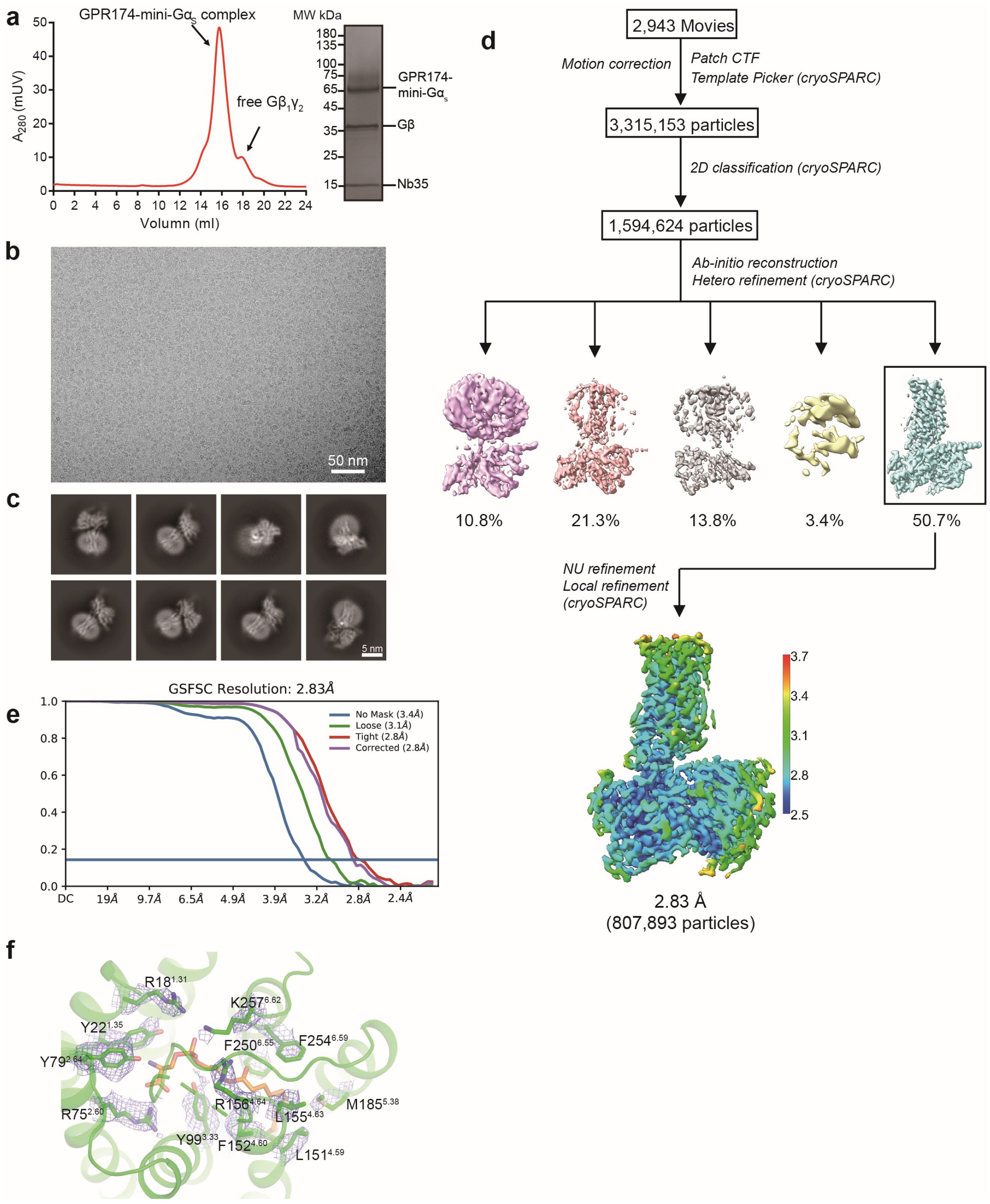

Supplementary Fig. 2 | Cryo-EM structure determination for the GPR174–G_s complex.

a, Size exclusion chromatography and SDS-PAGE analysis of the GPR174–mini-$G\alpha_s$/Gβγ/Nb35 complex.

b, Representative micrographs.

c, Representative 2D class averages.

d, Cryo-EM workflow chart.

e, Gold-standard FSC curves.

f, Representative cryo-EM maps for residues involved in binding lysoPS in GPR174.

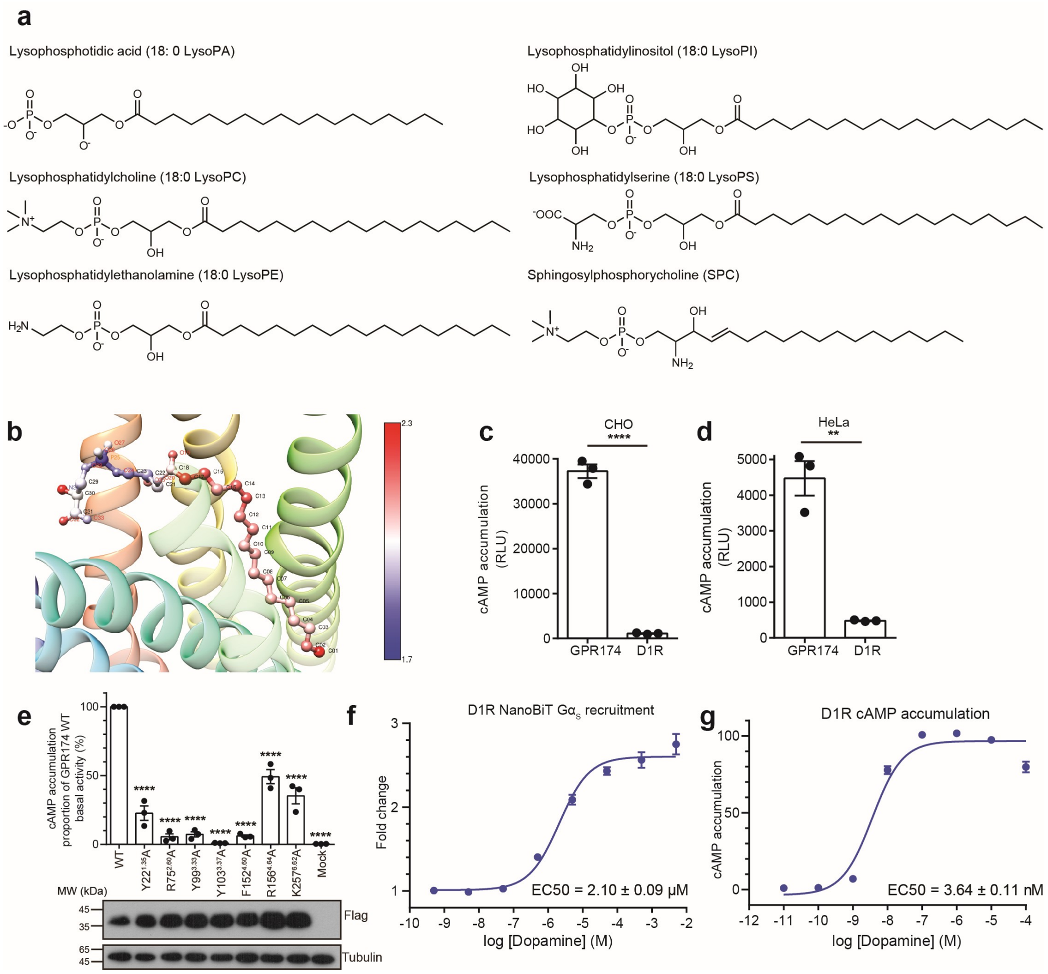

Supplementary Fig. 3 | Specific binding of GPR174 by lysoPS leads to high basal activity.

a, Chemical structure of LPs. For glycerophospholipids, the acyl chain is shown as stearic acid (18:0) at the sn-1 position of glycerol.

b, The root-mean-square-fluctuation (RMSF) of atoms in lysoPS when bound to GPR174 during 100-ns MD simulations. RMSF values from 1.7 to 2.3 Å are colored from blue to red.

c-d, cAMP accumulation levels in CHO cells (**b**) or HeLa cells (**c**) transfected with GPR174 or D1R. Bar graphs represent mean ± SEM from three independent experiments. Source data are provided as a Source Data file. *P* value for D1R versus GPR174 is < 0.0001 in CHO cells, and 0.0012 in HeLa cells.

e, The basal activity of GPR174 mutants determined by cAMP accumulation assay. The cAMP levels released by GPR174 mutants are expressed as percentage of WT. Statistical analysis was performed from three independent experiments using one-way ANOVA method (*****P* < 0.0001). All *P* values for GPR174 mutants versus WT are < 0.0001. The expression levels of mutants were determined by western blot. Bar graphs represent mean ± SEM from three independent experiments. Source data are provided as a Source Data file.

f-g, Dose response curves of D1R treated with increasing concentration of dopamine using NanoBiT Gα_s recruitment assay (**f**) and cAMP accumulation assay (**g**). Bar graphs represent mean ± SEM from three independent experiments. Source data are provided as a Source Data file.

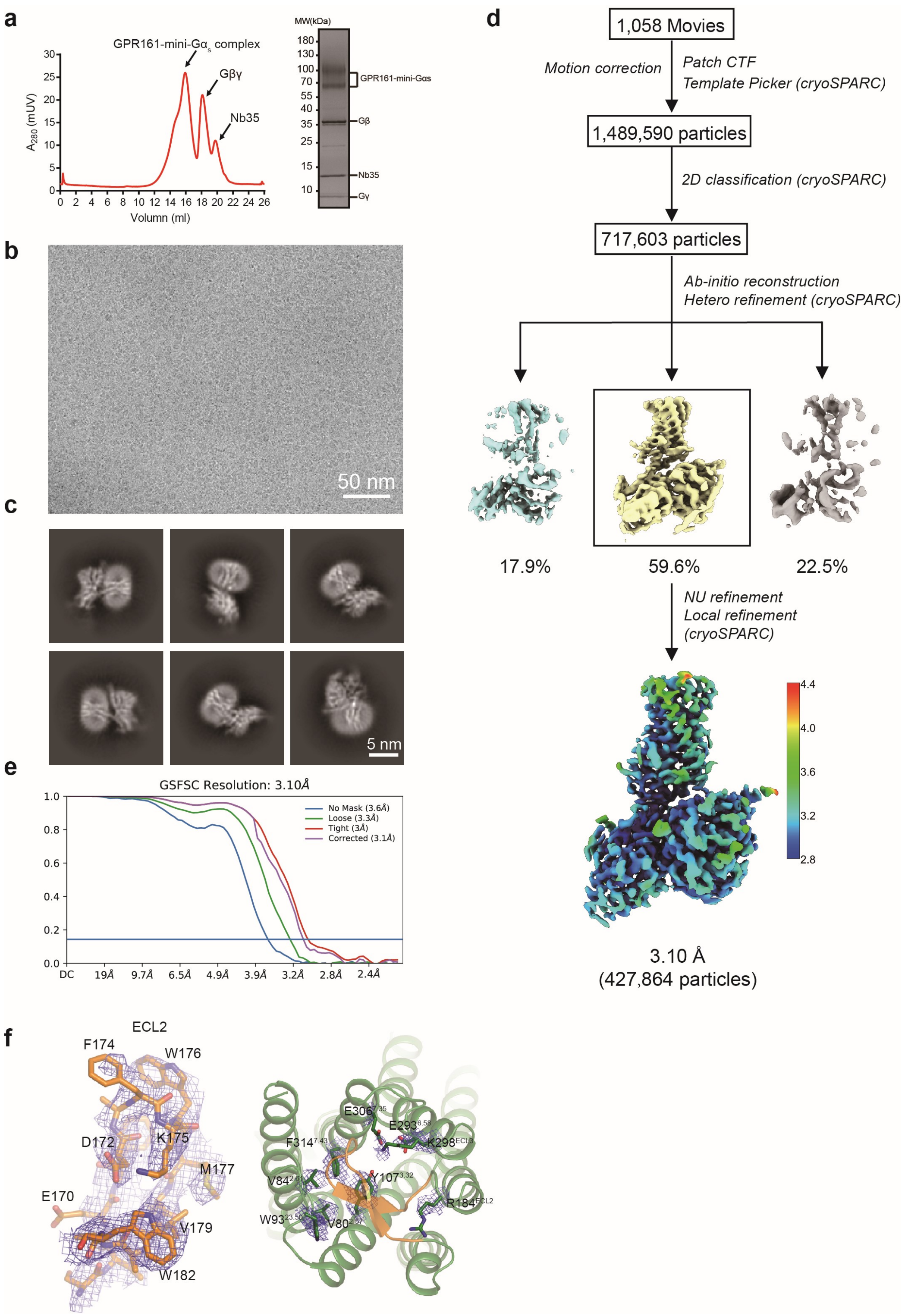

Supplementary Fig. 4 | Cryo-EM structure determination of the GPR161–G_s complex.

a, Size exclusion chromatography and SDS-PAGE analysis of the GPR161–mini-$G\alpha_s$/Gβγ/Nb35 complex.

b, Representative micrographs.

c, Representative 2D class averages.

d, Cryo-EM workflow chart.

e, Gold-standard FSC curves.

f, Representative cryo-EM maps for ECL2 and residues involved in binding ECL2.

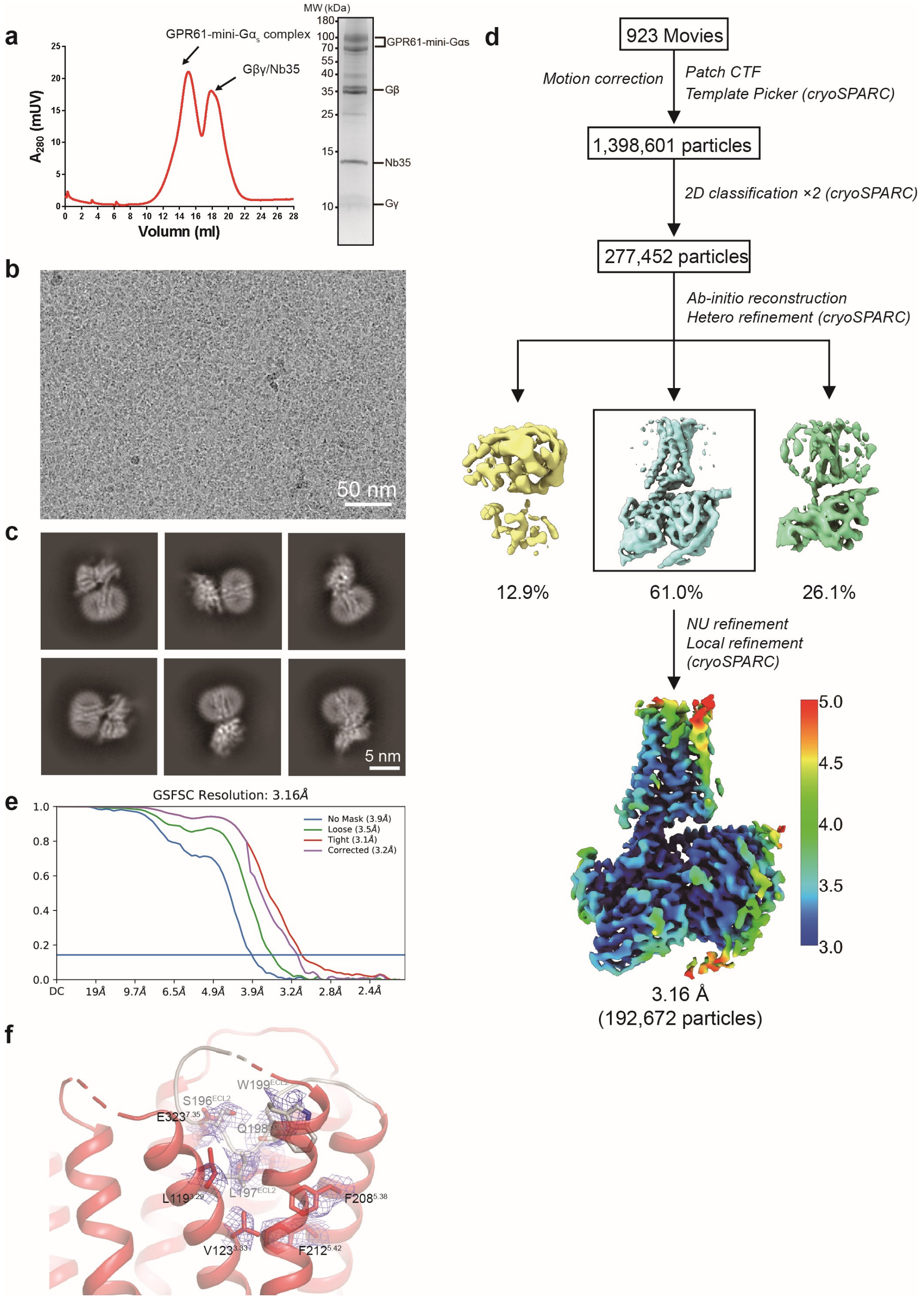

Supplementary Fig. 5 | Cryo-EM structure determination of the GPR61–G_s complex.

a, Size exclusion chromatography and SDS-PAGE analysis of the GPR61–mini-$G\alpha_s$/Gβγ/Nb35 complex.

b, Representative micrographs.

c, Representative 2D class averages.

d, Cryo-EM workflow chart.

e, Gold-standard FSC curves.

f, Representative cryo-EM maps for GPR61.

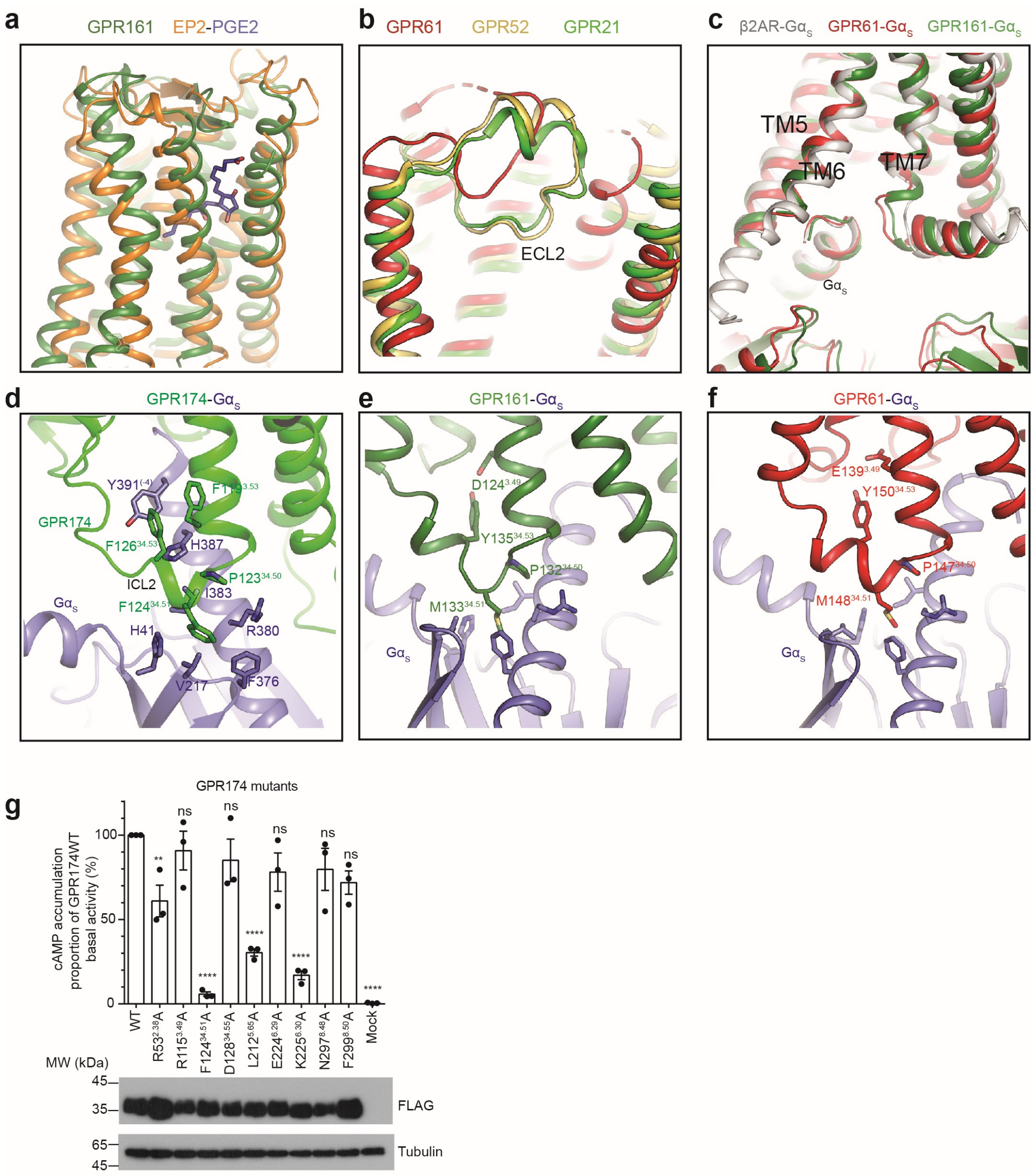

Supplementary Fig. 6 | Structural features of GPR61-G$_s$, GPR161-G$_s$ and GPR174-G$_s$ complexes.

a, Superposition of structures of GPR161 and PGE2-bound prostaglandin receptor EP2 (PDB: 7CX2) reveals a conserved β-hairpin structure of ECL2.

b, ECL2 in GPR61, GPR52 and GPR21 form a short loop structure.

c, Summary of EC_{50} and E_{max} of GPR174 mutants. Values are determined from three independent experiments.

d-f, GPR174–G$_s$ **(d)**, GPR161–G$_s$ (**e**) and GPR61–G$_s$ (**f**) show a common structural feature, where a hydrophobic residue is inserted into a hydrophobic pocket of the Ras domain of Gα$_s$.

g, Effects of mutations in GPR174 on its basal activity. The basal activities of GPR174 mutants are normalized as percentage of WT. The expression levels of GPR174 mutants are determined by western blot. Bar graphs represent mean ± SEM from three

independent experiments. *P* values for GPR174 mutants versus WT are 0.0187, 0.9756, < 0.0001, 0.7453, < 0.0001, 0.3501, < 0.0001, 0.4295, 0.1365, < 0.0001 (from left to right). Source data are provided as a Source Data file.

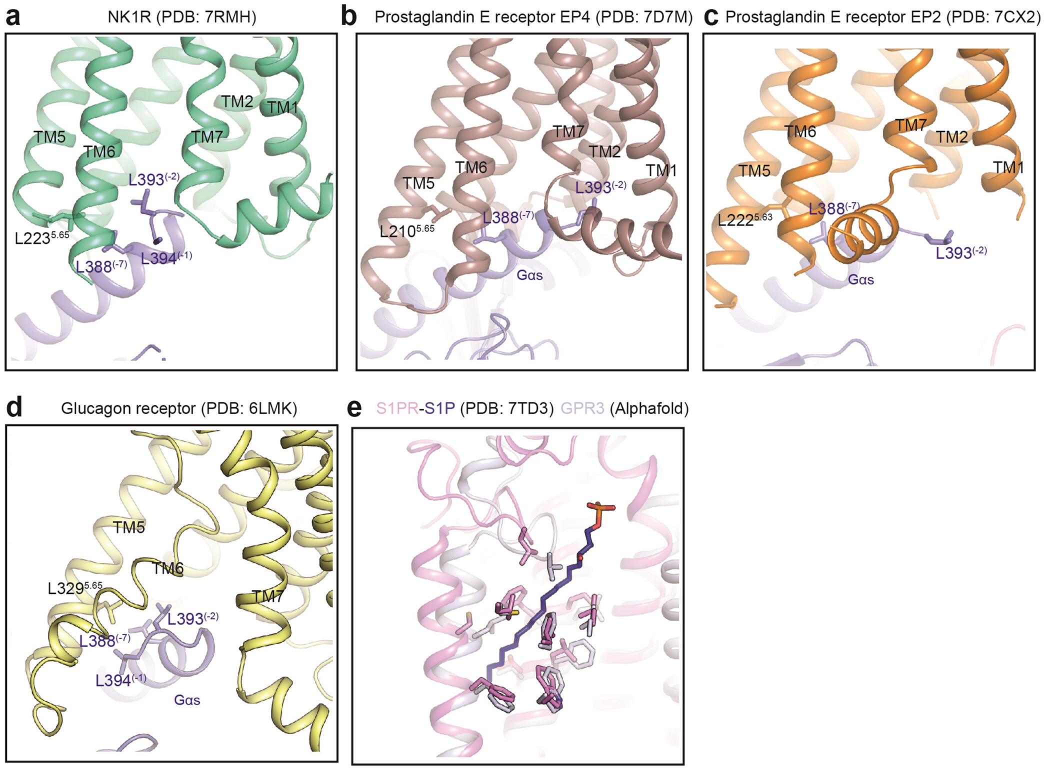

Supplementary Fig. 7 | Structural features of the non-canonical G_s coupling mode.

a-c, The NK1R (**a**), prostaglandin E receptor EP4 (**b**) and EP2 (**c**) adopt a non-canonical G_s coupling, where the C-terminal hook of $G\alpha_s$ is distorted.

d, A sharp kink was induced in the in the middle of TM6 at the glucagon receptor to accommodate a large hydrophobic residue L329$^{5.65}$ when bound to G_s.

e, Comparison of structures of GPR3 predicted by Alphafold and S1P-bound S1PR (PDB: 7TD3) reveals a conserved pocket for binding the acyl chain of lipid.

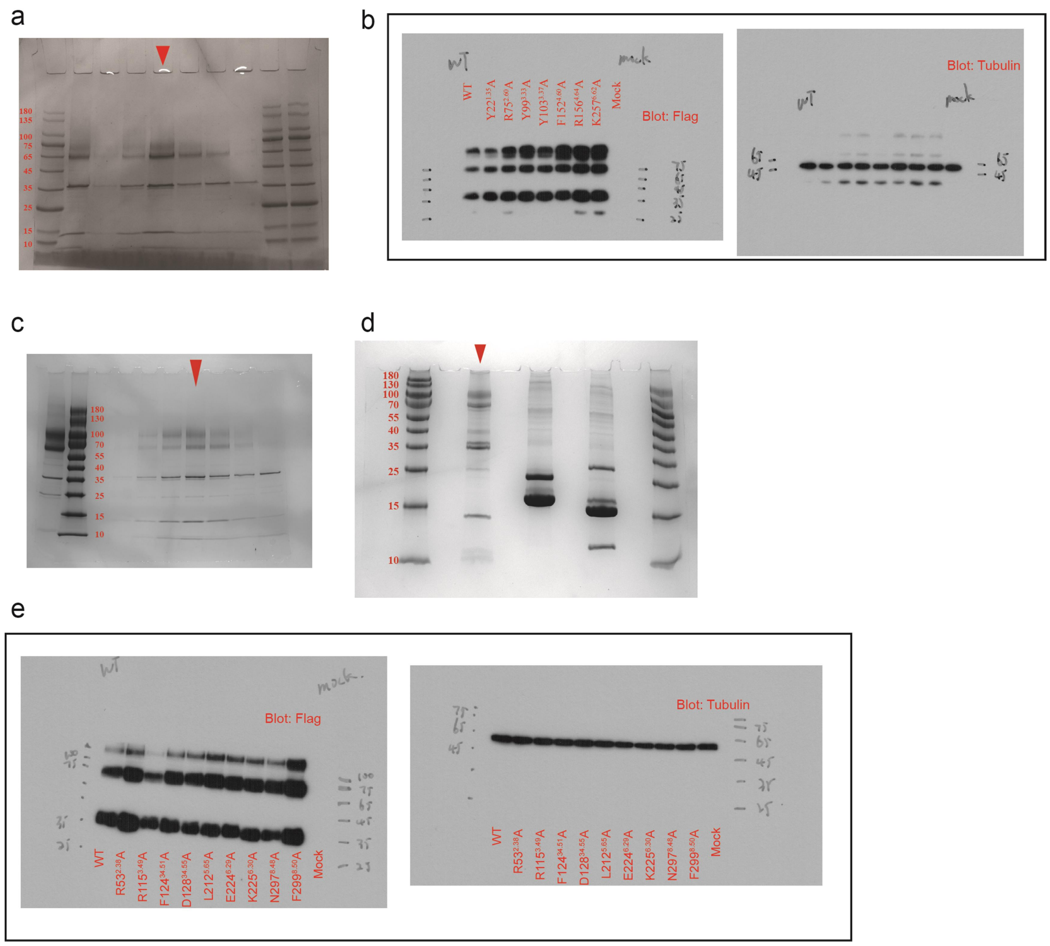

Supplementary Fig. 8 | Uncropped scans of western blots and SDS-PAGE gels.

a, Uncropped gel for Supplementary Fig. 2a.

b, Uncropped blots for Supplementary Fig. 3e.

c, Uncropped gel for Supplementary Fig. 4a.

d, Uncropped gel for Supplementary Fig. 5a.

e, Uncropped blots for Supplementary Fig. 6g.

Supplementary Table 1. Summary of the surface expression levels, the cAMP assay and NanoBiT mini-Gs recruitment assay for 81 oGPCRs and D1R. Receptors that show higher cAMP levels than others are shaded in yellow.

ID	Name	oGPCR Surface Expression levels	NanoBiT mini-Gαs recruitment Assay (RLU)	GloSensor cAMP accumulation assay (RLU)
1	GPR3	265022	473923	5021
2	GPR4	23681	217903	17006
3	GPR42	8482	86514	56
4	GPR6	292681	476606	24642
5	GPR12	65069	310438	7095
6	GPR15	6886	3207	80
7	GPR17	267345	49595	55
8	GPR19	151264	110332	91
9	GPR20	115107	51165	70
10	GPR21	145127	284368	1175
11	GPR22	107590	38173	74
12	GPR25	12495	70038	48
13	GPR26	4496	158641	29844
14	GPR27	37927	24160	55
15	GPR31	9507	62162	59
16	GPR32	70601	48919	78
17	GPR33	705566	84989	64
18	GPR34	1114515	78631	60
19	GPR35	260136	141970	97
20	GPR37	6034	61652	44
21	GPR37L1	1739	29240	71
22	GPR39	107047	123868	78
23	GPR45	90698	57837	54
24	GPR50	148815	52916	71
25	GPR52	749930	360098	10081
26	GPR61	66205	154099	1429
27	GPR62	6729	82697	254
28	GPR63	33316	47255	70
29	GPR65	14137	66254	98
30	GPR68	15127	59654	74
31	GPR75	687704	64163	91
32	GPR78	5317	255782	15127
33	GPR82	143098	30686	63
34	GPR83	3193	63914	42

35	GPR84	59935	30544	32
36	GPR85	130751	Not Detect	40
37	GPR87	59766	29095	54
38	GPR88	219186	48785	53
39	GPR101	635018	624968	15883
40	GPR132	137334	111872	86
41	GPR135	437608	205365	44
42	GPR139	230910	252656	48
43	GPR141	94513	101587	32
44	GPR142	10364	117544	57
45	GPR146	3052	14998	52
46	GPR148	16553	63390	72
47	GPR149	487876	57812	70
48	GPR150	282172	10819	60
49	GPR151	755184	88509	66
50	GPR152	44697	66819	107
51	GPR153	150812	31434	95
52	GPR160	10731	47989	83
53	GPR161	1332660	387058	978
54	GPR162	441509	69412	89
55	GPR171	278615	59087	91
56	GPR173	1427554	237927	147
57	GPR174	374110	420921	21630
58	GPR176	1862602	105220	122
59	GPR182	12965	4318	95
60	GPR183	526289	27552	112
61	MAS1	344644	66161	66
62	MAS1L	10116	6300	16
63	MRGPRD	171245	149022	140
64	MRGPRE	344531	72865	61
65	MRGPRF	558591	92389	59
66	MRGPRG	1553	4499	34
67	MRGPRX1	78853	15029	49
68	MRGPRX2	50248	37051	46
69	MRGPRX3	13064	72919	84
70	MRGPRX4	322176	108581	103
71	P2RY8	66598	109740	38
72	P2RY10	1796	6890	80
73	TAAR3	2142	40828	57
74	TAAR5	10876	8086	56
75	TAAR6	2987	10350	68
76	TAAR8	2977	23820	67
77	TAAR9	2876	20601	88

78	GPR1	743869	90157	71
79	GPR18	15127	117976	46
80	GPR55	447609	321778	115
81	GPR119	3852	122613	185
others	DRD1	459308	141484	130
others	pcDNA3.1	2364	Not Detect	74

Supplementary Table. 2 | Cryo-EM data collection, refinement and validation.

	GPR174-Gs (EMDB-37237) (PDB 8KH5)	GPR161-Gs (EMDB-37236) (PDB 8KH4)	GPR61-Gs (EMDB-37224) (PDB 8KGK)
**Data collection and processing**			
Magnification	64,000	64,000	64,000
Voltage (kV)	300	300	300
Electron exposure (e^{-}/Å^2)	50	50	50
Defocus range (μm)	1.39-2.78	0.48-2.56	0.87-2.48
Pixel size (Å)	1.08	1.08	1.08
Symmetry imposed	C1	C1	C1
Initial particle images (no.)	3,315,153	1,489,590	1,398,601
Final particle images (no.)	807,893	427,864	192,672
Map resolution (Å)	2.83 (masked)	3.10 (masked)	3.16 (masked)
FSC threshold	0.143	0.143	0.143
Map resolution range (Å)	2.5-3.6	2.8-4.4	3.0-5.0
**Refinement**			
Initial model used (PDB code)			
Model resolution (Å)	2.9	3.1	3.2
FSC threshold	0.143	0.143	0.143
Map sharpening *B* factor (Å^2)	-133.9	-152.2	-157
Model composition			
Non-hydrogen atoms	8208	7889	7810
Protein residues	1035	1009	1006
Ligands	2	0	0
B factors (Å^2)			
Protein	74.98	77.26	99.20
Ligand	71.73	-	-
R.m.s.d deviations			
Bond lengths (Å)	0.003	0.003	0.003
Bond angles (°)	0.565	0.664	0.555
Validation			
MolProbity score	1.70	1.77	1.69
Clashscore	10.58	13.46	11.23
Poor rotamers (%)	0.11	0.12	0.00
Ramachandran plot			
Favored (%)	97.16	97.28	97.37
Allowed (%)	2.84	2.72	2.63
Disallowed (%)	0.00	0.00	0.00

Supplementary Table 3 | Summary of EC_{50}, pEC_{50} and E_{max} for GPR174 mutants using the cAMP accumulation assay and NanoBiT G_s recruitment assay.

Values are determined from at least three independent experiments. ND, not determined due to poor response or poor fit.

GPR174 mutants + lysoPS (cAMP accumulation assay)			
GPR174	EC_{50}	pEC_{50} (M)	E_{max} (Proportion of GPR174WT basal activity, %)
WT	ND	ND	ND
Y22A	1.358 μM	5.87 ± 0.17	53.1 ± 3.2
R75A	ND	ND	ND
Y99A	71.6 nM	7.15 ± 0.10	69.7 ± 2.5
Y103A	ND	ND	ND
F152A	119.7 nM	6.92 ± 0.10	79.5 ± 3.0
R156A	ND	ND	ND
K257A	494.4 nM	6.31 ± 0.14	62.9 ± 2.7

GPR174 mutants + lysoPS NanoBiT G_s recruitment assay			
GPR174	EC_{50}	pEC_{50} (M)	E_{max} (proportion of basal activity, %)
WT	155.7 nM	6.81 ± 0.17	162.9 ± 3.5
Y22A	18.3 μM	4.74 ± 1.11	234.1 ± 197.7
R75A	ND	ND	ND
Y99A	1.054 μM	5.98 ± 0.18	155.8 ± 5.8
Y103A	ND	ND	ND
F152A	4.089 μM	5.39 ± 0.13	290.4 ± 19.8
R156A	ND	ND	ND
K257A	ND	ND	ND

GPR174 mutants + lysoPS (cAMP accumulation assay)			
GPR174	EC_{50}	pEC_{50} (M)	E_{max} (proportion of GPR174-Y99A maximum, %)
Y99A	71.6 nM	7.15 ± 0.10	100
Y99A/R53A	ND	ND	ND
Y99A/R115A	444.9 nM	6.35 ± 0.13	39.9 ± 2.1
Y99A/F124A	ND	ND	ND
Y99A/D128A	52.4 nM	7.28 ± 0.21	86.1 ± 5.6
Y99A/L212A	541.0 nM	6.27 ± 0.21	9.4 ± 0.9
Y99A/E224A	ND	ND	ND
Y99A/K225A	ND	ND	ND
Y99A/N297A	68.1 nM	7.17 ± 0.09	120.9 ± 3.9
Y99A/F299A	606.4 nM	6.22 ± 0.30	48.2 ± 7.3
