# Supplementary material for: Specific binding of GPR174 by endogenous lysophosphatidylserine leads to high constitutive Gs signaling
Source: Nat Commun. 2023 Sep 22;14:5901. doi: 10.1038/s41467-023-41654-3 (PMC10516915; doi:10.1038/s41467-023-41654-3)
Supplement: Supplementary file 3 — Reporting Summary [file 41467_2023_41654_MOESM3_ESM.pdf]

nature portfolio

Corresponding author(s): sanduo Zheng

Last updated by author(s): Aug 21, 2023

Reporting Summary

Nature Portfolio wishes to improve the reproducibility of the work that we publish. This form provides structure for consistency and transparency in reporting. For further information on Nature Portfolio policies, see our Editorial Policies and the Editorial Policy Checklist.

Statistics

For all statistical analyses, confirm that the following items are present in the figure legend, table legend, main text, or Methods section.

n/a	Confirmed	
☐	☒	The exact sample size (*n*) for each experimental group/condition, given as a discrete number and unit of measurement
☐	☒	A statement on whether measurements were taken from distinct samples or whether the same sample was measured repeatedly
☐	☒	The statistical test(s) used AND whether they are one- or two-sided<br>*Only common tests should be described solely by name; describe more complex techniques in the Methods section.*
☒	☐	A description of all covariates tested
☒	☐	A description of any assumptions or corrections, such as tests of normality and adjustment for multiple comparisons
☐	☒	A full description of the statistical parameters including central tendency (e.g. means) or other basic estimates (e.g. regression coefficient) AND variation (e.g. standard deviation) or associated estimates of uncertainty (e.g. confidence intervals)
☐	☒	For null hypothesis testing, the test statistic (e.g. *F*, *t*, *r*) with confidence intervals, effect sizes, degrees of freedom and *P* value noted<br>*Give P values as exact values whenever suitable.*
☒	☐	For Bayesian analysis, information on the choice of priors and Markov chain Monte Carlo settings
☒	☐	For hierarchical and complex designs, identification of the appropriate level for tests and full reporting of outcomes
☒	☐	Estimates of effect sizes (e.g. Cohen's *d*, Pearson's *r*), indicating how they were calculated

Our web collection on statistics for biologists contains articles on many of the points above.

Software and code

Policy information about availability of computer code

Data collection	cryoEM data was collected on Titan Krios G3 with detector Gatan or Falcon 4. Luminescent signal of NanoBit and cAMP accumulation assay were read in Tecan, Spark.
Data analysis	softwares applied for analysis were listed as: MotionCor2, cryoSPARC3.0, cryoSPARC3.3.1, Coot 0.9 pre, Graphpad Prism 7.0, Pymol 2.5.2, FlowJo 10, CHARMM36?CGenFF 2.5.1, GROMACS2021.4.

For manuscripts utilizing custom algorithms or software that are central to the research but not yet described in published literature, software must be made available to editors and reviewers. We strongly encourage code deposition in a community repository (e.g. GitHub). See the Nature Portfolio guidelines for submitting code & software for further information.

Data

Policy information about availability of data

All manuscripts must include a data availability statement. This statement should provide the following information, where applicable:

- Accession codes, unique identifiers, or web links for publicly available datasets
- A description of any restrictions on data availability
- For clinical datasets or third party data, please ensure that the statement adheres to our policy

The atomic structures have been deposited at the Protein Data Bank (PDB) under the accession codes 8KGK , 8KH4 and 8KH5. The EM maps have been deposited at the Electron Microscopy Data Bank (EMDB) under the accession numbers EMD-37224 , EMD-37236 and EMD-37237 . Source data are provided with this paper.

Research involving human participants, their data, or biological material

Policy information about studies with human participants or human data. See also policy information about sex, gender (identity/presentation), and sexual orientation and race, ethnicity and racism.

Reporting on sex and gender	not applicable
Reporting on race, ethnicity, or other socially relevant groupings	not applicable
Population characteristics	not applicable
Recruitment	not applicable
Ethics oversight	not applicable

Note that full information on the approval of the study protocol must also be provided in the manuscript.

Field-specific reporting

Please select the one below that is the best fit for your research. If you are not sure, read the appropriate sections before making your selection.

☒ Life sciences ☐ Behavioural & social sciences ☐ Ecological, evolutionary & environmental sciences

For a reference copy of the document with all sections, see nature.com/documents/nr-reporting-summary-flat.pdf

Life sciences study design

All studies must disclose on these points even when the disclosure is negative.

Sample size	For structural information, 2943 movies of GPR174-Gs, 1058 movies of GPR161, and 923 movies of GPR61 were collected and anlyzed as described in Methods.<br>For cell-based functional verification, NanoBit-based miniG recruitment assay, cAMP accumulation assay and NanoBit Gs recruitment assay, at least three biologically experiments were performed. Data were recorded and analyzed by fitting non-linear regression (dose-stimulation or dose-inhibition, three parameters) in GraphPad Prism 7.0. For cryo-EM structure determination, sample sizes were determined by the number of particles on EM grids sufficient to obtain an atomic structure. For cell-based assays, three independent measurement is enough to get reasonable comparison, to draw a firm conclusion. And sample sizes were determined based on the literature review and the number of independent experiments required for meaningful conclusions.<br>For cryo-EM structure determination, 807893, 427864 and 192672 pariticles were used to generate the final density map of GPR174-Gs, GPR161-Gs and GPR61-Gs complex, and result in a resolution of 2.83 Å, 3.10 Å and 3.16 Å, which is enough to for atomic model building. For cell-based assay, For cell-based assays, sample sizes were determined based on the literature review and the number of independent experiments required for meaningful conclusions.
Data exclusions	No data were excluded from the analyses.
Replication	Each experiment was reproduced at least three times on separate occasions. Experimental findings were reliably reproduced.
Randomization	The conclusion drawn from structure analysis and cell assay is not influenced by randomization of experiment, so sample were not randomized for the experiments.
Blinding	The conclusion drawn from structure analysis and cell assay is not influenced by the subjective judgment of the researchers, so no blinding was used in this study.

Reporting for specific materials, systems and methods

We require information from authors about some types of materials, experimental systems and methods used in many studies. Here, indicate whether each material, system or method listed is relevant to your study. If you are not sure if a list item applies to your research, read the appropriate section before selecting a response.

Materials & experimental systems

n/a	Involved in the study
☐	☒ Antibodies
☐	☒ Eukaryotic cell lines
☒	☐ Palaeontology and archaeology
☒	☐ Animals and other organisms
☒	☐ Clinical data
☒	☐ Dual use research of concern
☒	☐ Plants

Methods

n/a	Involved in the study
☒	☐ ChIP-seq
☒	☐ Flow cytometry
☒	☐ MRI-based neuroimaging

Antibodies

Antibodies used

M1 flag antibody used for agarose affinity purification were produced from hybridoma cell line. M2-Flag antibody (A8592) and anti α-Tubulin antibody (T6074) were from Sigma.

Validation

The M1 flag antibody has been used in multiple studies. https://www.sigmaaldrich.cn/CN/zh/product/sigma/f3040

Eukaryotic cell lines

Policy information about cell lines and Sex and Gender in Research

Cell line source(s)

Expi293F cells were purchased from Thermo Fisher Scientific. CHO and HeLa cells were gift from Guotai Xu's Lab in NIBS. Sf9 insect cell was acquired from Expression System Inc.

Authentication

All those cell lines were maintained by the supplier, no more authentication was performed by the authors.

Mycoplasma contamination

All cell lines mentioned were netative for Mycoplasma contamination.

Commonly misidentified lines
(See ICLAC register)

No commonly misidentified cell lines were used in this study.
